# Telehealth Impact in Frontier Critical Access Hospitals: Mixed Methods Evaluation

**DOI:** 10.2196/49591

**Published:** 2023-09-20

**Authors:** Saira Haque, Sydney DeStefano, Alison Banger, Regina Rutledge, Melissa Romaire

**Affiliations:** 1 RTI International Research Triangle Park, NC United States

**Keywords:** telehealth, virtual care, rural health, critical access hospital, access, telehealth impact, methods evaluation, mixed methods, thematic analysis, cost efficiency, telehealth cost, qualitative analysis, claims analysis

## Abstract

**Background:**

Frontier areas are sparsely populated counties in states where 65% of the counties have 6 or fewer residents per square mile. Residents access primary care at critical access hospitals (CAHs) located in these rural communities but must travel great distances for specialty care. Telehealth could address access challenges; however, there are barriers to broader use, including reimbursement and the need for practical implementation support. The Centers for Medicare & Medicaid Services implemented the Frontier Community Health Integration Project (FCHIP) Demonstration to assess the impact of telehealth payment change and technical assistance to adopt and sustainably use telehealth for CAHs treating Medicare fee-for-service patients in frontier regions.

**Objective:**

We evaluated the impact of the FCHIP Demonstration telehealth payment change and technical assistance on telehealth adoption and ongoing use using a mixed methods approach.

**Methods:**

We conducted a mixed methods evaluation of the 8 CAHs in Montana, Nevada, and North Dakota that participated in the FCHIP program. Key informant interviews and FCHIP program document review were conducted and analyzed using thematic analysis to understand how CAHs implemented their telehealth programs and the facilitators of program adoption and maintenance. Medicare fee-for-service claims were analyzed from August 2013 to July 2019 relative to a group of CAHs that did not participate in the demonstration project to understand the frequency of telehealth use for Medicare fee-for-service beneficiaries receiving care at the participating CAHs before and during the Demonstration program.

**Results:**

CAH staff noted several key factors for establishing and sustaining a telehealth program: clinical and administrative staff champions, infrastructure changes, training on telehealth processes, and establishing strong relationships with specialists at distant facilities to deliver telehealth services to patients of CAH. There was a modest increase in telehealth services billed to Medicare during the FCHIP Demonstration that were limited to a handful of CAHs.

**Conclusions:**

The frontier setting is characterized by a low population; and thus, the volumes of telehealth services provided in both the CAHs and comparison sites are low. Overall, CAHs reported that patient satisfaction was high and expressed the desire for more virtual services. Telehealth service selection was informed by perceived community needs and specialist availability. CAHs made infrastructure changes to support telehealth and expressed the desire for more virtual services. Implementation support services helped CAHs integrate telehealth into clinical and operational workflows. There was some increase in telehealth services billed to Medicare, but the volume billed was low and not enough to substantially improve hospital revenue. Future work to inform policy and practice could include standardized, formal community need assessments and assistance finding distant providers to meet those needs and further technical assistance around billing, service selection, and ongoing use to support sustainability.

## Introduction

### Background

Barriers to accessing health care for residents of rural communities have been widely recognized [1]. Frontier areas in particular have extremely low population density at great geographic distance from population centers and health care services [2]. Definitions of frontier used for state and federal programs vary. However, in general, frontier regions are sparsely populated rural areas based on geographical units such as county, zip code, or census tract. Considerations for these areas include physical distance and travel time to services, population density, relationships and associations with larger facilities, availability of paved roads, and seasonal variation in access to services. Common challenges in these areas include access to workforce, infrastructure, and local services and working within the realm of policies developed for more populated areas [2,3]. All of these make consistent access to health care challenging. Therefore, clinical providers, including critical access hospitals (CAHs), in frontier areas are especially challenged to provide timely high-quality care, particularly for specialty services, to their patients. CAHs are generally small (under 25 beds), are not located within 35 miles of another hospital, have a short length of stay (under 96 h), and are not resourced to provide a breadth of services [4]. Thus, people who live in an area served by a CAH may have to travel a great distance to access care. Technology-enabled health care delivery, or telehealth, has been widely touted as one possible answer to address challenges associated with specialist provider shortages in rural communities and significant travel distances between patients and providers [5-7].

In the last 15 years, telehealth has seen extraordinary growth, which exponentially increased during the coronavirus pandemic [6-8]. Medicare has historically limited telehealth use to certain providers in particular locations for certain conditions, yet uptake has been considerable [1] even before the pandemic. From 2006 through 2016, the number of telehealth visits grew almost 10-fold to an estimated 9.5 services per 1000 fee-for-service Medicare Part B enrollees, accounting for about US $27 million in Medicare fee-for-service payments [9]. However, in 2016, telehealth use was still infrequent compared to in-person visits, with 0.3% of all Medicare fee-for-service part B enrollees having a telehealth visit, and 2400 unique organizations billing Medicare for originating a telehealth visit [9].

Despite the growth in telehealth use over the last 15 years and policy changes that made it easier to deliver telehealth services to Medicare beneficiaries during the COVID-19 pandemic [10], barriers to telehealth adoption and ongoing use remain, particularly in rural communities. Barriers include limited access to reliable high-speed internet; lack of willingness among specialists to absorb the time and financial cost to become licensed to provide care via telehealth in another state or credentialed at another facility; skepticism that there will be a secure, reliable, and timely transfer of health information between rural health providers and distant providers (ie, the providers at a distant location who provide the clinical service via telehealth); concern about the efficacy of telehealth; long distances between a patient and the rural provider who can initiate a telehealth visit when home-initiated visits are not possible; significant upfront capital and training costs for telehealth equipment; and limited reimbursement that may not cover the staff time and equipment costs of originating a telehealth visit for a patient [11]. Fewer hospitals in rural areas have established telehealth programs compared to hospitals in more metropolitan areas [12], and one study found that only 35% of the smallest and often most rural hospitals, CAHs, had at least 1 telehealth program [13].

Paying particular attention to the reimbursement challenges, in 2016 the Centers for Medicare & Medicaid Services (CMS), in partnership with the Federal Office of Rural Health Policy at the Health Resources and Services Administration, launched the Frontier Community Health Integration Project (FCHIP) Demonstration. FCHIP was a 3-year project authorized under the Affordable Care Act of 2010 [14]; the demonstration changed certain regulations and Medicare fee-for-service reimbursement policies related to ambulance, skilled nursing, and telehealth services for CAHs in frontier regions of Montana, Nevada, and North Dakota.

For this demonstration, CMS defined frontier areas as those located in counties with 6 or fewer residents per square mile and located in states where 65% of the state’s counties have 6 or fewer residents per square mile. In total, 10 hospitals in 3 states were chosen to participate in the demonstration [15].

In total, 8 of the 10 hospitals (3 in Montana, 1 in North Dakota, and 4 in Nevada) participated in the telehealth component of the FCHIP Demonstration, which involved both a payment change and tailored one-on-one technical assistance to implement and grow a telehealth program. The intent of the payment change and technical assistance was to encourage increased use of telehealth by CAHs to improve health care access, especially for specialty care (Table 1) to potentially inform future directions.

**Table 1:** Characteristics of the FCHIP^a^ telehealth intervention.

	Before demonstration	During demonstration
FCHIP CAH^b^ facility payment	Under the Medicare physician fee schedule, a CAH serving as the originating site for a telehealth encounter is paid a fixed fee of about US $26.	A participating CAH could still bill for the originating site fee, and the CAH was reimbursed 101% of the cost for overhead, salaries, and fringe benefits, as well as the depreciation value of the telemedicine equipment.
Distant site provider payment	The distant site provider is paid according to the Medicare physician fee schedule for the service rendered.	No change
Telehealth modality	The telehealth modality is synchronous 2-way video technology.	No change
Technical assistance	Tailored technical assistance to implement telehealth was not provided.	HRSA^c^ funded one-on-one tailored technical assistance to help participating CAHs address telehealth implementation challenges. Technical assistance providers engaged in regularly scheduled calls and site visits with CAH administrators and clinical staff.

^a^FCHIP: Frontier Community Health Integration Project.

^b^CAH: critical access hospital.

^c^HRSA: Health Resources and Services Administration.

### Objectives

The purpose of this work is to identify whether changes in program implementation increased the use of telehealth among Medicare fee-for-service enrollees during the demonstration period (August 2016 through July 2019) relative to a predemonstration period (August 2013 through July 2016). We also sought to understand barriers and facilitators to telehealth implementation and use for participating CAHs. This study was designed to provide CMS with data on how the most rural hospitals that serve Medicare beneficiaries could stand up and sustain a telehealth program; the findings from these analyses also informed CMS and other federal policy makers in their decision-making about whether or not to sustain the programmatic changes made under the FCHIP Demonstration or adopt different policy changes for these very rural hospitals. This has implications not only for the demonstration program but also for the sustainability and ongoing use of telehealth services, particularly in rural areas. This also can help identify efforts to ameliorate the challenges for telehealth use in rural areas generally.

## Methods

### Study Design

We conducted a mixed methods evaluation that integrated perspectives on telehealth program implementation from CAH administrators and clinical staff with a trend analysis of Medicare fee-for-service telehealth claims. The qualitative piece was designed to uncover insights about telehealth implementation and use from program participants. These insights also provide the context around any observable trends in telehealth encounters for Medicare fee-for-service enrollees.

### Ethical Considerations

The Research Triangle Institute Institutional Review Board determined that this study was exempt from review because the evaluation of the demonstration was approved by CMS to examine changes in public programs [16].

### Qualitative Data Sources and Analyses

We conducted qualitative data collection and analysis of key informant interviews from FCHIP-participating CAH staff and review of program documents (ie, CAH’s applications to participate and their quarterly progress reports) submitted to CMS to understand the context around telehealth implementation. Individuals interviewed were identified by each participating FCHIP CAH as most knowledgeable about their telehealth programs. We developed semistructured interview guides to elicit information about implementation and use that were tailored to common stakeholder roles across participating CAHs. The questions in the interview guide were designed to elicit participant perspectives of the demonstration impact on 4 themes that related to the goals of the demonstration: hospital administration and infrastructure, hospital finances, access to health services, and regional or community-based spillover effects. Due to the small size of the CAHs, several individuals served in multiple roles (eg, providers and administrative staff), and we combined the interview guides accordingly in those cases. We conducted a total of 95 annual in-person or telephonic interviews with providers, hospital leaders, hospital administrative staff, and patients during 3 annual site visits from 2016 through 2019, which were recorded and transcribed. The first site visit was in-person, and the remaining 2 relied on telephone interviews. In addition, we reviewed program documentation that the CAHs provided as part of their participation in the demonstration and abstracted data from them. All of the documents FCHIP grantees were required to submit to CMS as part of their program participation were reviewed.

Transcripts from interviews and documents were uploaded into NVivo (QSR International), a software program that supports qualitative and mixed methods research [17] for analysis. The initial coding scheme was developed based on the goals of the demonstration to inform a blended inductive and deductive approach. The blended approach started with a deductive approach where the team started with a small set of codes based on the goals of the demonstration: hospital administration and infrastructure, hospital finances, access to health services, and regional or community-based spillover effects. The first set of documents and interviews were coded using this coding scheme. Then, the team met and reviewed the coding output (the passages flagged using the first set of general buckets) and updated and added to the coding scheme to more fully explicate topics, ideas, and patterns about barriers and facilitators to the implementation and use of telehealth using an inductive approach. The team created codes using the data and continued to refine the codebook during the analysis. The codebook included a list of codes and definitions. As coding continued, the team also added examples of when to use the codes or not to the codebook to help explicate decisions that were made. The team also identified quotes that could be used to explicate themes during this process. Two masters-trained analysts coded the documents with double coding of 10% of the documents. The entire team met biweekly to discuss the output and refine themes.

### Quantitative Data Sources and Analyses

We analyzed Medicare fee-for-service enrollment data and telehealth claims from 3 years before the demonstration (August 1, 2013, to July 31, 2016) to 3 years afterward (August 1, 2013, to July 31, 2019) from the Chronic Conditions Data Warehouse. These data include enrollment information that indicates the number of beneficiaries alive and enrolled in Medicare during the period and claims experience for each beneficiary. Claims denied by Medicare were excluded from the analysis. Claims data were assessed after 3 months of run-out for claims adjustments or revisions.

We conducted a descriptive analysis of counts and rates of telehealth services provided by CAHs each year during a predemonstration baseline period and during the 3-year demonstration period. The unit of analysis was the CAH identified using the CMS Certification Number. Counts and rates were calculated for each analytic year (August 1 through July 31). To compare service use across hospitals of varying sizes, we created rates of service use based on the number of Medicare beneficiaries who ever used a hospital of interest during the analytic year.

We compared the telehealth service use patterns among the participating CAHs (n=8 hospitals) with those of all other CAHs in Montana, Nevada, and North Dakota that did not participate in the demonstration and were also billed for telehealth services during the demonstration period (n=38 hospitals). The intent of this comparison is to place telehealth use among participating FCHIP Demonstration CAHs in the context of telehealth use among other CAHs in the 3 states; this comparison is not meant to infer any causal relationship between demonstration participation and changes in telehealth use. As such, we did not test for statistically significant differences in trends in telehealth use between demonstration CAHs and the other CAHs. Moreover, the sample of other CAHs was not designed to serve as a counterfactual for FCHIP CAHs, and there are key differences between the FCHIP CAHs and the other CAHs. First, the sample of other CAHs is not restricted to frontier CAHs. There are very few CAHs in areas designated as frontier regions in the 3 participating states. Those who were in these areas participated in the demonstration, so the other CAHs are located in rural but not necessarily frontier regions. Second, the expectation under this demonstration was that participating CAHs would be billing Medicare for telehealth services during the demonstration period of August 2016 to July 2019, so we wanted to compare rates of telehealth use in participating CAHs to CAHs that were also billing for telehealth during this same time. We did not require that other CAHs have the same telehealth Medicare billing patterns as FCHIP CAHs before the demonstration started.

The demographic characteristics of beneficiaries using the demonstration CAHs and other CAHs are similar (Table 2), despite the differences between groups in terms of geography (frontier vs nonfrontier). The CAHs primarily serve White, Medicare fee-for-service enrollees between 65 and 84 years of age, most of whom are enrolled in Medicare only.

**Table 2:** Sociodemographic characteristics of Medicare beneficiaries who ever used critical access hospital billing Medicare for telehealth in Montana, North Dakota, and Nevada during the demonstration period (August 1, 2016, to July 31, 2019).

Characteristic			Critical access hospitals		
			FCHIP^a^ telehealth critical access hospitals (n=8)		Other telehealth billing critical access hospitals in Montana, Nevada, and North Dakota (n=38)
**Age (years), n (%)**					
	<65	888 (14)		10,925 (12)	
	65-74	3109 (49)		46,698 (48)	
	75-84	1713 (27)		24,580 (27)	
	≥85	698 (11)		11,835 (13)	
White, n (%)			5836 (92)		85,576 (94)
Female, n (%)			3426 (54)		49,161 (54)
Dually eligible for Medicare and Medicaid, n (%)			1078 (17)		14,566 (16)
Total Medicare fee-for-service beneficiaries, n			6344		91,038

^a^FCHIP: Frontier Community Health Integration Project.

We examined 2 telehealth, Medicare fee-for-service claims-based outcomes of interest: the frequency of telehealth services rendered at the CAH and the type of specialty care provided by the distant provider via telehealth. An originating site telehealth visit was defined as any claim where the Current Procedural Terminology code was “Q3014.” We identified professional claims for the same beneficiaries on the same date of service as an originating telehealth visit and where the professional claim had a place of service code indicting telehealth and a procedure code approved by CMS for telehealth reimbursement to identify the specialties of the distant providers who rendered telehealth services to patients of CAH [18]. Once the professional claims were identified, we did a frequency analysis of the provider specialty listed on the claim.

## Results

### Telehealth Program Implementation

#### Overview

We identified several key themes that support the implementation and use of telehealth during the demonstration based on document reviews and interviews. Table 3 highlights the findings and how they relate to the initial coding scheme based on the goals of the demonstration.

**Table 3:** Impact of the demonstration on hospital administration and infrastructure, hospital finances, access to health services, and regional or community-based spillover effects.

Domain	Results
Hospital administration and infrastructure	Areas that were essential to facilitate a successful telehealth program: Administrative and clinical champions Training providers and staff on how to use telehealth services Building and maintaining relationships with distant site providers Technical assistance was well-received Topics for technical assistance included advertising or marketing in the community, telehealth-specific educational resources, billing support, and workflow analysis
Hospital finances	Overall volume of Medicare encounters was too low to have a substantial impact on hospital financial performance Mixed perceptions on whether or not cost-based reimbursement was adequate across CAHs^a^ Some participants noted that telehealth services were provided yet not consistently billed
Access to health services	Participating CAHs expressed concern about billing and reimbursement yet expected to maintain telehealth services after the demonstration 2 CAHs never billed Medicare for telehealth encounters during the demonstration yet reported providing services
Regional or community-based spillover effects	Community knowledge of telehealth varied prior to the demonstration according to participants CAHs marketed telehealth in the community and at other facilities to increase knowledge of services

^a^CAH: critical access hospital.

#### Clinical and Administrative Champions Are Essential to the Success of a Telehealth Program

CAH administrators were instrumental in securing necessary program resources like equipment and staff to coordinate telehealth visits with distant providers. They were also critical to ensuring that CAH staff appropriately billed Medicare for telehealth services rendered to Medicare fee-for-service enrollees so that the CAH received all available payments. Clinical provider champions, CAH physicians, and nurse practitioners who referred patients to telehealth services and shared experiences with telehealth throughout the organization modeled desired telehealth referral patterns and helped secure buy-in from practitioners who were hesitant to refer patients to specialty care via telehealth. One telehealth coordinator unscored the hesitancy to change that champions had to address

...it’s not part of their workflow and they don’t want to make it part of their workflow. It’s not how they’ve done it and they don’t want to change.

Clinical champions also helped design changes in workflow processes to ensure an efficient and seamless referral process for CAH staff and the patient. Turnover of hospital administrators and providers was common during the Demonstration. CAH staff observed that the impact of turnover was that telehealth-related activities often slowed or paused and restarting activities took time. This underscores the importance of consistent champions to program implementation.

#### Changes to Physical Infrastructure Were Needed to Accommodate Telehealth Visits

Although all participating CAHs reported having or recently having had some telehealth capabilities in place prior to the demonstration, they did not have widespread use and needed to address the physical infrastructure needed to conduct telehealth visits. During the first year of the demonstration, CAH staff focused on establishing dedicated physical locations for telehealth visits, obtaining the necessary video equipment, such as mobile telehealth carts, and acquiring robust, high-speed internet access that could support synchronous audio and video communication if they did not already have such internet access. As one CAH administrator noted, the demonstration was a pivotal part of infrastructure changes “It’s helped us beef up our staffing and we’ve been able to get equipment we wouldn’t otherwise have been able to spend money on if we didn’t have money to cover the staff.”

#### Training Providers on How to Use Telehealth Services Facilitated More Referrals for Telehealth

CAH administrators and telehealth coordinators observed that most requests for telehealth came from CAH providers who thought a patient needed to see a specialist, but they noted that many providers erroneously believed that telehealth was an on-demand service. Therefore, CAH staff, with the support of one-on-one technical assistance and staff provided through the demonstration, developed training programs that educated CAH staff on which specialists were available via telehealth and when telehealth was appropriate versus when a face-to-face visit was preferred, how to refer to a particular specialist, and how to follow-up with the specialist and the patient after the telehealth visit to ensure coordination and continuity of care. Administrators observed that once clinical staff were well-trained on the process and expectations, they were more likely to refer patients to care using telehealth.

CAH staff also shared anecdotes of patients’ satisfaction with the telehealth encounter and appreciation for the fact that the patient did not have to travel for a specialist appointment. As one telehealth coordinator noted,

Absolutely, we have to [continue telehealth services]. Our patients have come to rely on it, and I know a great number of people who would simply not get services if we didn’t have telehealth.

Patients and caregivers noted their appreciation of having access to telehealth services. One patient said

Generally, I have a schedule once every 3 months with my nephrologist in [...] which is about 350 miles one way from here... ...For me the drive to [...] is difficult. I’m not much of a driver anymore because of health problems [...], and I can’t drive by myself [...] It doesn’t sound like a lot, but you have to go the day before and generally come back the day after. So, that’s 3 days out of your work week. For me it was heaven sent to have the telemed[icine] set up, and I love it. I only have to drive 14 miles to [FCHIP CAH] to do [the telehealth visit] there.

CAH administrators also observed that their clinical staff became more willing to refer patients for telehealth services when they heard of the positive experiences other patients were having.

#### Building a Good Relationship With Distant Site Providers Takes Time and Effort but Is Critical to the Success of a Telehealth Program

To successfully schedule telehealth appointments, engage in timely follow-up of specialists’ recommendations, and handle billing and insurance, CAH and distant site provider staff noted the importance of frequent and effective communication. At the start of the demonstration, most participating CAHs spent time establishing relationships with distant site providers, such as large in-state hospitals or clinics, but as the demonstration progressed, CAH staff focused on improving the referral and follow-up processes to ensure a more satisfying experience for the patient, the CAH staff, and the distant provider. As one telehealth coordinator noted,

Technology is a big part of it [telehealth], but for us it’s a relationship business. We’ve worked hard over the years to build the relationships and select people who want to do this [telehealth] and are good at it...The biggest thing I can say is to develop relationships with these folks.

#### Cost-Based Reimbursement Was Generally Well-Received, but Telehealth Visits Were Too Infrequent for Cost-Based Reimbursement to Have a Significant Impact on Hospital Finances

Reimbursement for telehealth increased during the demonstration because payment was based on costs incurred. While CAH staff uniformly appreciated this change, almost all staff noted that the volume of Medicare fee-for-service telehealth visits was too low for the payment change to noticeably increase hospital revenue.

### Claims-Based Analysis of Telehealth Use

Prior to demonstration implementation, only 1 of the 8 participating CAHs had billed Medicare for telehealth encounters. Despite participating in the FCHIP Demonstration, only 6 of the 8 participating CAHs billed Medicare for telehealth during the demonstration period, resulting in a total of 289 unique Medicare fee-for-service telehealth encounters. Of the 289 unique telehealth encounters that originated at a demonstration CAH, 79% (n=228) of encounters were linked to a corresponding professional claim of the distant site provider. We found that the specialties most commonly used included physical medicine and rehabilitation (n=58, 20%), cardiology (n=49, 17%), nurse practitioners (n=46, 16%), nephrology (n=43, 15%), and mental health (n=23, 8%). Other specialties included radiology (n=9, 3%), oncology (n=6, 2%), and other specialties such as endocrinology, gastroenterology, neurology, and gerontology (n=26, 9%).

The 289 Medicare fee-for-service telehealth encounters were associated with 150 unique telehealth users, two-thirds of whom used telehealth once. A smaller percentage of this group used telehealth repeatedly during the demonstration, with 15% using telehealth twice, 7% using telehealth 3 times, and 12% using telehealth 4 or more times.

**Figure 1:**
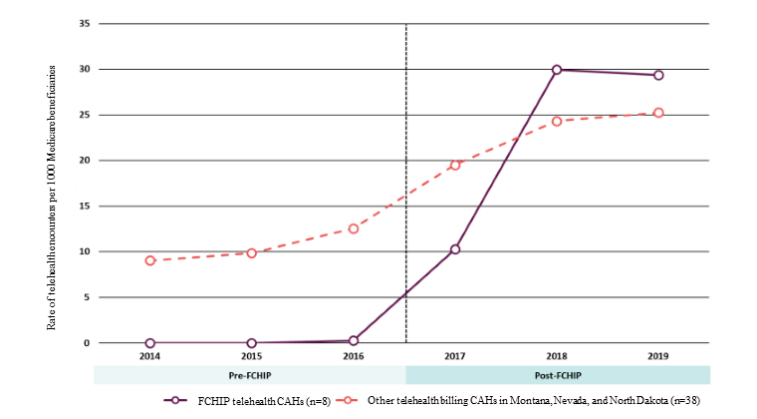
Annual rate of telehealth encounters per 1000 Medicare fee-for-service beneficiaries among FCHIP Demonstration CAHs and other CAHs, 2014-2019. CAH: critical access hospital; FCHIP: Frontier Community Health Integration Project.

Demonstration CAHs were expected to bill for more telehealth encounters for Medicare enrollees during the demonstration, and the telehealth encounter rate among demonstration CAHs did increase in 2017 before plateauing in 2018 and 2019, and the other CAHs showed a similar pattern over time (Figure 1).

## Discussion

### Principal Findings

Implementing telehealth in rural communities takes significant time, effort, and commitment. Technical assistance helped CAHs integrate telehealth into clinical and operational workflows, and organization support and physical infrastructure changes were needed to implement and sustain services. Collaboration between originating and distant sites was also a critical factor in the success of the demonstration CAH telehealth programs; good collaboration improves referral processes, information exchange, and care coordination. However, access to care can only be improved to the extent that a distant site clinic or health system has the specialties of interest available to provide services and they have the specialists willing to provide telehealth services for patients of CAH. Because CAHs do not have a large patient volume, the CAHs found it more challenging to negotiate with distant site providers as to what telehealth services the distant site will offer the patients of CAH. Some used existing relationships as part of a system, while others used regional services available to rural providers. As a result, the specific specialty needs of some patients of CAH were not necessarily addressed because the CAH was not able to secure certain types of specialty care via telehealth. Future work could include standardized, formal community need assessments and assistance finding distant providers to meet those needs.

While there was an increase in billed telehealth encounters for Medicare fee-for-service beneficiaries during the demonstration, the number of encounters was still quite low and plateaued in the last 2 years of the demonstration. Low volumes mean that cost-based reimbursement alone will likely not be enough to persuade a CAH to introduce or sustain a telehealth program. The lack of significant annual growth in telehealth encounters among the FCHIP CAHs was not wholly unexpected based on key informant interviews. Several CAH administrators shared that some, but not all, of their providers were willing to refer patients to telehealth, which may have reduced the number of telehealth encounters scheduled and subsequently hosted by the FCHIP CAHs. Moreover, in these very rural communities, the number of Medicare fee-for-service beneficiaries receiving care at the CAH is low, and even fewer are in need of specialty services that are well-suited to telehealth. During the short demonstration period, CAHs were not able to significantly increase demand for telehealth, but when there was demand, CAH administrators noted that it was often for a specialist with whom the CAH did not have an established relationship. As discussed earlier, securing the right mix of specialists willing to engage in telehealth is a challenge for the CAHs, but CAH administrators were hopeful that over time they would be able to grow the list of specialists with whom they partnered, thereby increasing demand for telehealth.

Despite challenges in program development, CAHs reported that satisfaction was high among patients who did use telehealth services, and all participating CAHs expressed the desire to be able to refer their patients for even more specialty services through telehealth. CAHs also reported that they planned to continue with telehealth after the demonstration period was over.

There are several limitations to note with this analysis. This study was not designed to support a causal inference between demonstration activities and telehealth use. We sought to obtain a range of perspectives of staff at the participating CAHs, but there may be other perspectives. For the qualitative analysis, the primary data sources were key informant interviews and program documentation. Although the goal of the interviews was to gain feedback, including viewpoints, from a variety of stakeholders, they were identified by the CAH. Thus, there is no guarantee that the individuals who participated in the interviews are representative of the entire staff of the FCHIP CAHs. In addition, these were often the same people who completed the program documentation, so implementation information described in program documents may also not fully represent the view of all FCHIP CAH staff.

Analyses of telehealth use among Medicare beneficiaries were limited to billed telehealth encounters, and some CAH staff indicated that they were not billing for all services delivered due to a lack of experience billing Medicare for telehealth or due to perceptions that the reimbursement amount was not worth the time to bill. Therefore, Medicare telehealth use reported here is undercounted. In addition, to construct rates for the hospital analysis, we assigned beneficiaries to CAHs based on using the hospital at least once during the analytic year. Beneficiaries could receive services at more than one CAH in a single analytic year. While our examination of this limitation found that occurrences were extremely rare, it is important to note the possibility.

Sample sizes for claims analyses were small because, in any given community in which the FCHIP CAHs reside, the number of Medicare beneficiaries receiving telehealth services is small. Therefore, the claims analyses were underpowered to detect statistically significant changes in use from one year to the next. Thus, the results should be interpreted as descriptive.

The participating sites operate within a localized context, and thus, the applicability outside of Montana, Nevada, and North Dakota must be considered. However, the considerations about considering the workforce available, proximity to other resources, and the needs of the individuals in their communities could be applied more broadly outside of those states.

Notably, this study occurred before the COVID-19 pandemic, over which time, Medicare eased telehealth policy and billing rules to support rapid telehealth uptake and ease the burden on health care organizations [18]. These changes included expanded reimbursement for services such as removing restrictions on the locations where the patient and providers could be located, expanding the services that could be reimbursed via telehealth, and promoting payment parity. Services that were added included speech, occupational, and physical therapy, substance use disorder treatment, and home dialysis. In addition, the types of providers who could deliver telehealth services and be reimbursed, such as occupational and speech therapists, were expanded during the pandemic. Some states also expanded their participation in the Interstate Medical Licensure Compact to allow for service provision across state lines during the pandemic [19]. The federal government also provided support for broadband during the pandemic to support areas that were not served by high-speed internet [20].

Despite these changes to accommodate the move to telehealth during the public health emergency, some of the considerations identified by the demonstration remain and can continue to help inform telehealth efforts and policy-making specific to rural communities. In particular, service selection, ongoing financial and regulatory considerations, and evaluation and continuous improvement are ongoing considerations [21,22]. In addition, this work focused on real-time video visits rather than the broader scope of virtual care. The considerations for sustainability and ongoing use identified for real-time video visits may be applicable for organizations as they plan for ongoing use of virtual care. During the demonstration, service selection was largely driven by the availability of distant providers. Similarly, during the COVID-19 pandemic, service selection was driven by what could become a virtual service quickly and not necessarily on the needs of the community or on patient preferences. However, going forward, service selection may be driven by other factors such as a community assessment or patient needs [23,24].

Similarly, both the demonstration and the COVID-19 public health emergency represented temporary changes in financial considerations around telehealth. Monitoring changes and billing can be a challenge, as indicated by the CAHs. The payer landscape continues to change, and going forward, CAHs and other health care organizations will need to be able to monitor changes and address and consider financial and regulatory considerations as part of their ongoing virtual care strategy [25,26]. CAHs also indicated that they appreciated the technical assistance they received through the demonstration, which points to a role of technical support in helping health care organizations interpret the changing landscape of telehealth regulation and reimbursement.

This work represents an evaluation of telehealth over 3 years, and a longer-term evaluation was not completed. Future directions might include assessing outcomes over a longer period of time and using the results of these assessments to inform telehealth strategies for telehealth uptake and ongoing use. In 2021, the demonstration was extended for several more years to allow for pre- and postpandemic analyses of telehealth use at participating CAHs. Additional knowledge gaps that warrant future study include understanding how frontier communities and providers could identify and prioritize telehealth services such as conducting a standardized community needs assessment to identify the most pressing needs for specialty care delivered via telehealth and identifying useful parameters for deciding whether to deliver specialty care in-person or virtually via telehealth. Aspects to consider for ongoing telehealth service selection include identifying the needs of the community, severity of the patient, number of patients lost to follow-up, and the importance of an in-person examination.

### Conclusions

Telehealth has a great promise to help ameliorate the challenges of health care provision in rural areas, and reimbursement is a well-recognized limitation to telehealth uptake and sustainability. However, changing telehealth reimbursement policy is not sufficient for maintaining a successful, sustainable telehealth program. Important considerations related to telehealth service selection that emerged from this work include identifying and maintaining connections with distant sites that provide specialty services and engaging clinician buy-in on the use of telehealth for different services and parameters for face-to-face versus virtual visits. Administrative considerations include establishing clear and consistent telehealth referral processes, addressing staffing and infrastructure challenges, licensing, credentialing, and developing ways to monitor and address reimbursement and regulatory changes. CAHs have fewer staff and infrastructure to address these challenges and may benefit from additional support to sustain telehealth services and address barriers to care for their patient populations.

## Abbreviations

CAH: critical access hospital
CMS: Centers for Medicare & Medicaid Services
FCHIP: Frontier Community Health Integration Project

## Data Availability

The quantitative data sets generated and analyzed during this study are not publicly available because the work was performed under contract for the Centers for Medicare & Medicaid Services (CMS), and as such, CMS owns the data. Others with access to the CMS Chronic Conditions Data Warehouse could use the same methodology to set up similar claims analyses. The qualitative data are not publicly available. However, the corresponding author can discuss with CMS making quantitative and qualitative data available for reasonable requests.
